# Innovative Use of Bleomycin Electrosclerotherapy (BEST) for High-Flow Arteriovenous Malformations in the Head District: Preliminary Results of Two Cases

**DOI:** 10.3390/jcm14072516

**Published:** 2025-04-07

**Authors:** Linda Latini, Sandra Bracco, Samuele Cioni, Sara Leonini, Flavia Cascino, Paolo Gennaro

**Affiliations:** 1 Maxillofacial Surgery Operative Unit, Department of Mental Health and Sense Organs, Santa Maria Le Scotte, University Hospital of Siena, 53100 Siena, Italy; latilinda94@gmail.com (L.L.); flaviacascino@hotmail.com (F.C.); 2 Neuroimaging and Neurointervention Unit, Santa Maria Le Scotte, University Hospital of Siena, 53100 Siena, Italy; sandrabracco64@gmail.com (S.B.); s_cioni3@yahoo.it (S.C.); s_leonini@yahoo.it (S.L.)

**Keywords:** AVM, vascular lesions, vascular malformations, bleomycin, electrosclerotherapy

## Abstract

**Background**: According to the ISSVA 2018 classification, arteriovenous malformations (AVMs) are high-flow vascular malformations, distinct from low-flow lesions. About 60% of extracranial AVMs occur in the head and neck, making their management a focus of maxillofacial surgery. Due to their complexity, precise diagnosis and careful treatment planning are crucial for optimal aesthetics and structural preservation. The standard approach combines embolization with surgical resection, though Bleomycin electrosclerotherapy (BEST) has recently gained recognition. **Methods**: From July 2023 to December 2024, a total of 16 patients with vascular malformations were treated with bleomycin electrosclerotherapy at the Azienda Ospedaliera Universitaria Senese (AOUS). Among them, two patients were affected by arteriovenous malformations. These two patients underwent this treatment to avoid more invasive and demolitive procedures, considering the anatomical region involved. Both patients had previously been treated at other hospitals, experiencing subsequent lesion recurrence. Preoperative evaluation included angiographic and ultrasound studies. The patients underwent electrosclerotherapy sessions and were closely monitored during follow-up. The uniqueness of this innovative approach lies in the use of fractionated doses of bleomycin for each treatment session, compared to the standard protocols described in the literature. **Results**: BEST has demonstrated efficacy in the treatment of high-flow AVMs by delivering bleomycin into the interstitial tissue and subsequently applying electroporation so the drug’s effects can be precisely localized and amplified. The macroscopically evident results, patient satisfaction, and, most importantly, the objective ultrasound flow data demonstrate the effectiveness of this treatment. **Conclusions**: Arteriovenous malformations (AVMs) pose treatment challenges due to their variability and lack of standardized guidelines. This study explores electrosclerotherapy with bleomycin in two head and neck AVM cases, using fractionated doses to enhance safety and efficacy. The findings support its potential as a minimally invasive alternative, warranting further research on broader applications.

## 1. Introduction

Arteriovenous malformations (AVMs) are classified as vascular malformations according to the latest classification by the International Society for the Study of Vascular Anomalies (ISSVA) (2018) [1]. They belong to high-flow malformations, along with arteriovenous fistulas, and are distinct from low-flow malformations, such as venous, lymphatic, and capillary anomalies. AVMs are congenital, meaning they are present from birth, even if they are not immediately visible. This suggests that their etiology is inherently genetic. Several studies have investigated the genetic pathways implicated in AVM formation. Although the molecular mechanisms remain incompletely understood, the most frequently implicated pathways include TGF-β, Ras/MAPK, and PI3K/AKT [2]. A key distinguishing factor between AVMs and arteriovenous fistulas is their pathogenic origin. While both are high-flow vascular anomalies, arteriovenous fistulas are typically acquired, often resulting from trauma. According to recent epidemiological data, the annual incidence (per 100,000 individuals) of vascular anomalies is estimated to be 9.85 for venous malformations, 1.48 for capillary malformations, 2.31 for arteriovenous malformations, 0.24 for lymphatic malformations, and 5.82 for other types [3]. AVMs predominantly affect the head and neck region in 47.4% of cases, followed by the extremities (28.5%) [4]. A recent large-scale study further categorized head and neck AVMs based on their anatomical distribution: 18.1% in the cheeks, 15.1% in the ears, 9% in the forehead, 8% in the periorbital region, 8% in the nose, 5% in the upper lip, 3.4% in the lower lip, 3.2% in the scalp, and 2.9% in the mandible [5]. Given this anatomical predilection, maxillofacial surgeons frequently play a key role in both the resective and reconstructive management of these lesions. The clinical presentation of AVMs alone is often insufficient for an accurate diagnosis, as they frequently manifest as soft-tissue swellings of variable size and depth, reddish or bluish flat lesions with a benign appearance, or ulcerated, destructive, and hemorrhagic lesions [6]. A hallmark feature distinguishing AVMs from low-flow malformations is the presence of palpable pulsations. Patients, particularly those with head and neck involvement, often report sensations of buzzing or whooshing, especially when the lesion is compressed at rest. Unlike venous malformations, AVMs do not exhibit volume changes with body position or movement [7]. Among the most severe complications, the risk of life-threatening hemorrhage is a major concern due to the high-flow nature of these malformations, which can result in uncontrolled, high-pressure bleeding that is difficult to manage [8]. Additionally, the chronic and progressive nature of AVMs, coupled with frequent mismanagement and lack of standardized treatment protocols, often results in significant psychological distress for patients, who are frequently referred between multiple centers without clear therapeutic guidance [9]. If left untreated, AVMs can also lead to systemic complications, including high-output cardiac failure [10]. Several classification systems have been developed for AVMs. The Shobinger classification is based on clinical staging, ranging from quiescent Stage I to decompensated Stage IV [11]. This system is particularly useful for assessing disease progression, as it underscores the inherent evolution and destructive potential of AVMs. Current evidence suggests that AVMs exhibit a continuous growth tendency, ruling out the possibility of spontaneous regression. Furthermore, external triggers, such as trauma, and internal factors, such as hormonal fluctuations during pregnancy or the menstrual cycle, can exacerbate their progression [12]. The Yakes classification, in contrast, is based on angioarchitecture, categorizing AVMs according to the vascular structures involved, thereby guiding endovascular treatment strategies [13,14]. The SECg classification integrates clinical features, endovascular characteristics, and growth rates, offering a comprehensive approach that includes a potential treatment algorithm. This classification system considers key factors such as lesion location, symptomatology, complications, vascular architecture, and growth dynamics [15].

Currently, no universally accepted treatment guidelines exist for AVMs, and their management remains an area of active investigation. A multidisciplinary approach is essential, involving maxillofacial surgeons, interventional radiologists, anesthesiologists, and geneticists to develop personalized treatment plans that incorporate emerging therapeutic options and technologies [16]. A wide range of therapeutic strategies have been described in the literature. Early techniques, such as simple arterial ligation, have been largely abandoned due to their tendency to exacerbate disease progression by promoting collateral vessel recruitment. Embolization alone is often reserved for emergency cases, whereas combined approaches integrating embolization with surgical resection have emerged as the standard of care, offering improved outcomes. Sclerotherapy, historically one of the first treatment modalities for vascular anomalies, remains a widely used option for both low-flow and high-flow lesions. Various sclerosing agents have been employed, with bleomycin emerging as a promising alternative in recent years. The combination of bleomycin with electroporation—a technique originally developed for oncological applications—has demonstrated efficacy and safety across multiple types of vascular anomalies, including AVMs [17]. As a non-invasive approach, it represents a viable alternative to more aggressive interventions, particularly when it achieves satisfactory outcomes while preserving surrounding structures. In this study, the authors present preliminary results from the treatment of two patients with AVMs using bleomycin electrosclerotherapy (BEST). The favorable outcomes observed support the potential of this technique as a valuable therapeutic option, allowing for effective disease control while avoiding more invasive procedures whenever possible.

## 2. Materials and Methods

Between July 2023 and December 2024, the authors treated 16 patients with vascular malformations using electrosclerotherapy with bleomycin. The study was approved by the Institutional Review Board of AOUS (Azienda Ospedaliera Universitaria Senese), and all patients provided informed consent in accordance with the Declaration of Helsinki. Each patient was fully informed about the off-label nature of the treatment and consented to both the procedure and the publication of their clinical data. Inclusion criteria consisted of adult patients diagnosed with an arteriovenous malformation (AVM) in the head and neck region. Exclusion criteria included the presence of other types of vascular malformations, eligibility for alternative and more effective treatments, documented allergies to bleomycin, and a history of pulmonary disease. Table 1 summarizes the clinical data of the two AVM cases.

Following the initial consultation at our clinic, during which the clinical characteristics of the malformations were thoroughly evaluated, both patients underwent contrast-enhanced and non-contrast MRI of the head and neck, as well as angiography. Although angiography is not universally recommended as a first-line diagnostic tool according to current guidelines, we included it in our diagnostic workflow to obtain a comprehensive understanding of the malformation’s angioarchitecture, thereby optimizing treatment planning. After completing the imaging assessments, both cases were reviewed by a multidisciplinary team of maxillofacial surgeons, neuroradiologists, and anesthesiologists. This collaborative approach allowed for the formulation of an individualized treatment strategy. Before the initial procedure, both patients underwent a chest X-ray to rule out pre-existing pulmonary conditions and establish a baseline reference for potential pulmonary complications during treatment. Before each session, Doppler ultrasound evaluations were conducted to assess hemodynamic parameters, including systolic peak velocity, diastolic peak velocity, and resistance index (RI). The systolic peak velocity reflects the maximum blood flow during cardiac contraction, while the diastolic peak velocity indicates perfusion during relaxation. The RI, derived from these measurements, provides an objective quantification of vascular resistance. These parameters are essential for monitoring disease progression and assessing therapeutic response. To ensure consistency and minimize inter-operator variability, all ultrasound examinations were performed by the same operator (S.L.), given the operator-dependent nature of this imaging modality. Electrosclerotherapy with bleomycin was selected as the treatment modality due to the anatomical complexity of the AVM locations, which would have necessitated highly destructive surgical interventions with preoperative embolization, often leading to suboptimal aesthetic and functional outcomes. In particular, AVMs affecting the columella and lower lip pose significant reconstructive challenges, with a high risk of cosmetic and functional impairment even in the hands of experienced surgeons. Given these considerations, a personalized treatment protocol was deemed the most appropriate approach.

The proposed protocol involved the administration of bleomycin at a maximum dose of 15 mg per session, with up to six sessions at this dosage, for a total cumulative dose of 90 mg. The Cliniporator device (IGEA, Carpi, Mo, Italy) is an advanced electroporation system that generates high-intensity electric fields for targeted tissue treatment. It delivers eight short-duration (100 μs) electric pulses at an intensity of 1000 V/cm through six steel needle electrodes. These electrodes are mounted on an insulated plastic support, forming a specialized applicator known as the “finger electrode”. This wearable configuration allows for precise placement on the tissue, creating a localized and intense electric field that induces electroporation of cell membranes within the targeted area. All procedures were conducted under general anesthesia due to the significant pain associated with electroporation, which is poorly tolerated, particularly in the head and neck region. Despite the brief duration of each session and the mild burning sensation induced by bleomycin injection, the primary source of discomfort arises from the application of electric pulses. To ensure treatment consistency and facilitate the development of a standardized protocol, electrosclerotherapy was performed by the same operators throughout the study (L.L., S.C., F.C.). Electroporation was applied transcutaneously in patient 1 and transmucosally in patient 2. Typically, each application involved electric pulses of 1000 V/cm, with the number of pulses adjusted based on lesion size and extent. To mitigate potential adverse effects such as hyperpigmentation in friction-prone areas, the authors, in collaboration with the nursing staff, established an intraoperative and postoperative management protocol. This protocol included strict avoidance of adhesive bandages both intraoperatively and postoperatively. Additionally, immediate local application of ice was recommended to reduce postoperative edema and provide symptomatic relief. Each treatment session required a maximum hospital stay of one night, though in most cases, hospitalization was unnecessary, and the procedure was performed on an outpatient basis due to its favorable safety profile and low morbidity. Sessions were spaced approximately two months apart to allow for optimal therapeutic response. Post-treatment follow-up included weekly in-person evaluations for the first month. Thereafter, patients were instructed to send weekly photographs to assess lesion progression, accompanied by a report detailing any complications or side effects.

## 3. Results

Among the 16 patients treated, 14 presented with low-flow malformations, including 11 venous malformations (VMs) and 3 capillary malformations (CMs), while 2 were diagnosed with AVMs. Both AVM patients were women, aged 37 and 51, with a clinical history tracing back to childhood when their malformations were initially asymptomatic, appearing only as small reddish patches. Both reported a significant increase in lesion size and worsening symptoms following pregnancy. They sought treatment after experiencing multiple unsuccessful interventions. Patient 1 had previously used unspecified topical creams and both systemic and topical anti-angiogenic drugs, while Patient 2 had undergone embolization procedures that ultimately exacerbated her condition. Both patients had a history of recurrent bleeding episodes, including severe hemorrhages requiring emergency care. Additionally, they reported psychological distress, including difficulties in accepting their condition and anxiety-depressive symptoms driven by insecurity, fear, and the absence of effective treatment options. Following treatment with bleomycin electrosclerotherapy, both patients achieved favorable clinical outcomes, with cessation of bleeding and a reduction in vascular flow indices on ultrasound. The immediate resolution of bleeding after the first session was particularly significant, as it markedly improved both their quality of life and psychological well-being. The administered bleomycin doses per session remained below the maximum recommended 15 mg, leading to a total cumulative dose substantially lower than the literature-reported threshold. This fractionated approach allowed for a gradual and controlled therapeutic effect, with the flexibility to extend treatment if needed, particularly for extensive AVMs or in cases of recurrence. Given the satisfactory outcomes obtained within a limited number of sessions, the treatment protocol was adjusted accordingly, prioritizing efficacy while minimizing potential side effects. Additionally, this approach preserved the possibility of future retreatment if necessary, ensuring a tailored and adaptable therapeutic plan. The interval between sessions was individualized based on each patient’s clinical response. When feasible, a transmucosal rather than a transcutaneous electroporation technique was preferred to prevent skin-related adverse effects such as hyperpigmentation and peau d’orange changes. This strategy was particularly viable in the head and neck region, where access via the oral or nasal mucosa is often possible. Table 2 reports the results for both patients.

The follow-up period of at least six months included clinical evaluation, ultrasound assessment, and head MRI with and without contrast. Although a diagnostic angiography was performed preoperatively, the authors decided not to repeat this examination postoperatively. As it is an invasive procedure and not well tolerated by patients, they chose not to include it in the standard follow-up protocol, reserving its use only in cases of suspected recurrence. Fortunately, it was not needed for either of the two patients.

The authors regard the outcomes as highly satisfactory, particularly considering the minimally invasive nature of the procedure. In Case 1, treatment was temporarily suspended to allow for ongoing clinical and ultrasound monitoring, facilitating the early detection of potential pathological recurrences or anomalous vascular flows that might indicate the need for additional sessions. In Case 2, despite achieving excellent therapeutic results, an additional treatment session was scheduled, as further improvement was deemed feasible. No intraoperative bleeding was observed, and blood loss remained negligible across all treatment sessions. Neither patient developed major post-procedural complications, such as pulmonary fibrosis, cutaneous hyperpigmentation, or flagellated erythema. However, Patient 2 experienced notable edema and multiple mucosal aphthous lesions following the first session, which resolved spontaneously within approximately two weeks. No pharmacological intervention was required, except for the application of an analgesic gel for symptomatic relief of the ulcers.

**Figure 1:**
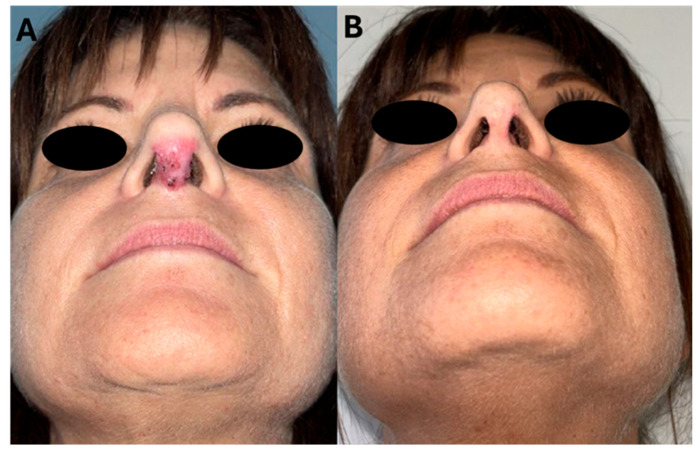
Patient 1: (**A**) Pre-operative appearance; (**B**) post-operative appearance.

**Figure 2:**
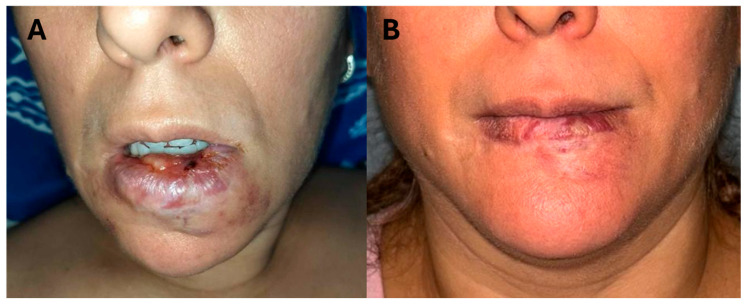
Patient 2: (**A**) Pre-operative appearance; (**B**) post-operative appearance.

Figure 1 and Figure 2 show the pre-operative and post-operative appearance of Patient 1 and Patient 2, respectively.

## 4. Discussion

The authors present the preliminary outcomes of two patients with arteriovenous malformations (AVMs) who had previously undergone unsuccessful treatments at other institutions and were subsequently managed with electrosclerotherapy using bleomycin. This approach offers a minimally invasive alternative with promising therapeutic results. AVMs, along with arteriovenous fistulas, represent high-flow vascular malformations that differ significantly from low-flow lesions such as venous malformations. AVMs are congenital malformations resulting from genetic mutations that are not yet fully elucidated. Ongoing research in the literature aims to clarify the pathogenic mechanisms underlying AVMs, and novel minimally invasive diagnostic techniques, such as liquid biopsy, have been developed [18].

The management of AVMs remains a significant clinical challenge due to the absence of universally accepted treatment guidelines. A multidisciplinary approach is essential to ensure comprehensive and effective management through collaboration among specialists from various disciplines [19]. The inherent characteristics of AVMs present therapeutic difficulties. Their high-flow dynamics render these lesions highly unstable, and even minor trauma can precipitate severe, life-threatening hemorrhages [20]. For this reason, early intervention is recommended, even for small, asymptomatic lesions. A watchful waiting approach with clinical follow-up alone may not be appropriate, as treatment is more feasible and effective before the lesion progresses to an ulcerated or hemorrhagic state. Another major challenge in AVM management is the high recurrence rate when treatment is incomplete. Inadequate intervention can stimulate the formation of collateral vessels, leading to more aggressive and treatment-resistant recurrences [21]. The concept of radicality in vascular malformations is complex and difficult to define. Unlike oncologic resections, where standardized margins are well established, vascular lesions lack universally recognized dimensional criteria or macroscopic margins, complicating treatment planning and increasing the risk of recurrence. Over the years, various therapeutic strategies have been proposed and evaluated. Treatments associated with excessive bleeding risk, such as isolated surgical excision or ligation of the feeding artery, are now largely abandoned. These interventions have been shown to exacerbate AVMs by promoting the formation of new feeders and increasing lesion size and aggressiveness [4,22]. Embolization is a widely used approach, particularly for the emergency management of hemorrhagic AVMs [23]. A variety of embolic agents have been developed, each with distinct properties that facilitate endovascular occlusion via different access routes [24]. However, embolization alone is generally considered a palliative rather than curative treatment, as it provides temporary stabilization without addressing the underlying pathology [25]. A combination of embolization and surgery has emerged as a preferred approach for extracranial AVMs, particularly in the head and neck region. This staged strategy typically involves initial embolization to devascularize the lesion and minimize intraoperative bleeding, followed by surgical resection [26]. The interval between these two phases can range from days to weeks [27]. Based on the authors’ experience, a single-stage approach combining embolization and resection under the same anesthesia may be advantageous, as it optimizes lesion control while reducing the need for multiple interventions. After excision, the resulting defect can be closed primarily in cases where skin elasticity permits or reconstructed using local, regional, or distant flaps for larger defects [28]. Sclerotherapy has demonstrated high success rates in the management of vascular anomalies. Historically, ethanol has been the most widely used sclerosant due to its potent endothelial ablation effect, which often leads to complete lesion eradication with low recurrence rates. However, ethanol is associated with a high risk of complications [29,30], including cutaneous necrosis, site infections, systemic toxicity, and nerve injury [31]. In recent years, bleomycin has gained attention as a promising alternative sclerosant.

Originally developed as an antibiotic [32,33], its antitumor properties were soon recognized, leading to its FDA approval in 1975 for the treatment of squamous cell carcinoma, testicular carcinoma, and malignant lymphoma [34,35]. Due to its sclerosing properties, bleomycin has also been studied for vascular anomalies, though its use in this context remains off-label. Initially applied to low-flow malformations, intralesional injection of bleomycin induces endothelial damage and vessel fibrosis, resulting in lesion regression [36,37].

The treatment of high-flow lesions presented challenges due to rapid washout of the sclerosant. However, interstitial injections surrounding the lesion have enabled circumferential sclerosis, yielding promising results in early-stage, small AVMs [38,39]. A major advancement in AVM therapy has been the introduction of electroporation-assisted sclerotherapy, termed electrosclerotherapy (BEST).

Electroporation is a biophysical technique that transiently increases cell membrane permeability through the application of electrical pulses, allowing enhanced intracellular uptake of therapeutic agents. Additionally, electroporation induces localized vasoconstriction, which helps retain bleomycin at the target site while minimizing intra-procedural bleeding. Electroporation has been widely adopted in oncology under the term electrochemotherapy (ECT), where it enhances the cytotoxic effects of chemotherapeutic agents, including bleomycin [40]. BEST has demonstrated significant efficacy in treating both low-flow and high-flow vascular anomalies by combining the sclerosing action of bleomycin with the targeted permeability enhancement of electroporation, leading to a more effective and controlled therapeutic outcome [41,42]. As described by Colletti et al., BEST can also be integrated into a surgical treatment strategy. In large, surgically challenging lesions, preoperative BEST can facilitate lesion downstaging, allowing for less aggressive resection. Postoperative BEST can be employed to target residual nidus and stabilize surgical outcomes [43]. For all these reasons, the authors believe that BEST treatment for AVMs should be considered, particularly for lesions in the head and neck region. This area includes many regions that are challenging to manage surgically, making a less invasive yet equally effective alternative highly valuable. Both small lesions, which could potentially be treated effectively with just a few sessions, and large lesions, which could be downstaged with bleomycin for subsequent surgical intervention, are eligible for this treatment. Additionally, with our fractional approach, even larger lesions could potentially be completely managed with bleomycin in multiple sessions. Despite its advantages, BEST is not exempt from recurrence risk, particularly given the difficulty in defining precise AVM margins. Potential complications associated with bleomycin include flagellated erythema, cutaneous hyperpigmentation—particularly in areas subject to repeated friction—and pulmonary fibrosis. However, the doses used in vascular malformation protocols remain well below the threshold associated with pulmonary toxicity, supporting its favorable safety profile. To minimize skin-related side effects, specific intraoperative and postoperative precautions should be implemented. These include minimizing injection site trauma, avoiding mechanical friction, and restricting the use of adhesive dressings. Also, patient education regarding potential complications is crucial to ensure early recognition and appropriate management.

Despite the encouraging results of this study, the authors acknowledge the limitations of a small patient cohort. Larger studies with extended follow-up are required to validate the efficacy and safety of BEST in AVM management. Further research is also needed to optimize treatment protocols, including ideal bleomycin dosage and electroporation parameters, and to elucidate the molecular mechanisms underlying this therapeutic approach.

If confirmed by future studies, BEST could represent a paradigm shift in AVM treatment, providing a minimally invasive alternative to traditional surgical resection while preserving functional and aesthetic outcomes. The authors hope that these findings will encourage multicenter clinical trials and interdisciplinary collaborations to refine and standardize BEST as a first-line treatment for AVMs.

## 5. Conclusions

Arteriovenous malformations (AVMs) are challenging to treat due to their variability and the lack of standardized guidelines. Electrosclerotherapy with bleomycin, combining sclerosing effects with electroporation, has shown promise as an alternative approach. This study presents two cases of head and neck AVMs in patients unsuitable for surgery and embolization due to anatomical constraints. Using lower bleomycin doses per session, the total 90 mg dose was distributed over multiple treatments, ensuring a controlled, minimally invasive approach with reduced side effects. The technique proved safe and effective, offering a flexible protocol adaptable to both early-stage and larger AVMs. This pilot study aims to serve as a starting point for recruiting more patients to make the research more significant and to investigate the large-scale effects of bleomycin at fractionated and personalized doses. The goal is to assess both its efficacy and potential side effects, allowing for the development of protocols that minimize the risk of recurrence.

## Figures and Tables

**Table 1:** Clinical data of the two AVM patients.

	Vascular Malformation	Age	Sex	Localization	Pre-Operative Dimensions	Symptoms	Onset of Symptoms	Previous Treatment	Shobinger Classification
Patient 1	AVM	51	Female	Nasal Columella	2 × 2 cm	Bleeding, Ulceration	During the Pregnancy	Antiangiogenetics Drugs	III
Patient 2	AVM	37	Female	Inferior Lip	5 × 3 cm	Bleeding, Ulceration	During the Pregnancy	Embolizations	III

**Table 2:** Results.

	Number of Session	Dose per Session (mg)	Cumulative Dose (mg)	First Measurement of the Systolic Peak	Last Measurement of the Systolic Peak	First Measurement of the IR	Last Measurement of the IR	Post-Operative Dimensions	Follow Up
Patient 1	3	15 + 1, 5 + 1, 5	18	41.9 cm/s	30.7 cm/s	0.8	0.5	0 × 0 cm	9 months
Patient 2	2	3, 75 + 2, 25	6	43.3 cm/s	26.2 cm/s	0.60	0.45	2 × 1 cm	6 months

## Data Availability

The raw data supporting the conclusions of this article will be made available by the authors on request.

## Abbreviations

The following abbreviations are used in this manuscript:

AVMs	ArteroVenous Malformations
VMs	Venous Malformations
LMs	Lymphatic Malformations
CMs	Capillary Malformations
